# Utilizing Heart Rate Variability for Coaching Athletes During and After Viral Infection: A Case Report in an Elite Endurance Athlete

**DOI:** 10.3389/fspor.2021.612782

**Published:** 2021-09-03

**Authors:** Laura Hottenrott, Thomas Gronwald, Kuno Hottenrott, Thimo Wiewelhove, Alexander Ferrauti

**Affiliations:** ^1^Department of Training and Exercise Science, Faculty of Sports Science, Ruhr University Bochum, Bochum, Germany; ^2^Department of Performance, Neuroscience, Therapy and Health, Faculty of Health Sciences, Medical School Hamburg, Hamburg, Germany; ^3^Institute of Sports Science, Department of Training Science and Sports Medicine, Martin-Luther-University Halle-Wittenberg, Halle, Germany

**Keywords:** orthostatic test, athlete monitoring, heart rate variability, viral infection, return to sport, endurance sport, cardiac autonomic control, marathon

## Abstract

**Background:** Viral diseases have different individual progressions and can lead to considerable risks/long-term consequences. Therefore, it is not suitable to give general recommendations on a time off from training for athletes. This case report aims to investigate the relevance of detecting heart rate (HR) and HR variability (HRV) during an orthostatic test (OT) to monitor the progression and recovery process during and after a viral disease in an elite endurance athlete.

**Methods:** A 30-year-old elite marathon runner contracted a viral infection (upper respiratory tract infection) 4 weeks after a marathon race. RR intervals in HR time series in supine and standing positions were monitored daily in the morning. Analyzed parameters included HR, the time-domain HRV parameter root mean square of successive difference (RMSSD), peak HR (HRpeak) in a standing position, and the time to HR peak (tHRpeak).

**Results:** During the 6-day viral infection period, HR increased significantly by an average of 11 bpm in the supine position and by 22 bpm in the standing position. In addition, the RMSSD decreased from 20.8 to 4.2 ms, the HRpeak decreased by 13 bpm, and the tHRpeak increased by 18 s in the standing position significantly. There were no significant changes in the pre-viral infection RMSSD values in the supine position. The viral infection led to a significant change in HR and HRV parameters. The cardiac autonomic system reacted more sensitively in the standing position compared to the supine position after a viral infection in the present case study.

**Conclusion:** These data have provided supportive rationale as to why the OT with a change from supine to standing body position and the detection of different indicators based on HR and a vagal driven time-domain HRV parameter (RMSSD) is likely to be useful to detect viral diseases early on when implemented in daily routine. Given the case study nature of the findings, future research has to be conducted to investigate whether the use of the OT might be able to offer an innovative, non-invasive, and time-efficient possibility to detect and evaluate the health status of (elite endurance) athletes.

## Introduction

With the pandemic spread of the novel coronavirus (SARS-CoV-2) that causes coronavirus disease 2019 (COVID-19; World Health Organization, 2020), healthcare professionals and coaches are faced with an increasing number of athletes seeking advice on when and how to restart training after recovery from viral diseases. This is challenging, as practical and evidence-based recommendations for a return to sports after infectious episodes are limited and heterogeneous. Preliminary approaches regarding COVID-19 provide symptom-based decision schemes to make recommendations for additional assessment and intervention measures (Nieß et al., 2020; Schnellhorn et al., 2020). It is the aim of this case report to investigate the relevance of detecting heart rate (HR) and HR variability (HRV) during an orthostatic test (OT) to monitor the progression and recovery process during and after a viral disease in an elite endurance athlete.

Due to the negative effects on health status and performance capacity, viral infections in sports must be detected early and physical exercise is not recommended in the case of a viral infection (Roberts, 1986). Especially, myocarditis is a significant cause of sudden cardiac death and sudden cardiac arrest (SCD/SCA) in athletes (Harmon et al., 2016) with case series reporting myocarditis as a potential source of SCD/SCA in up to 8% (Halle et al., 2020). In this regard, it is important to find very early indicators and sensitive markers (ideally before the onset of symptoms) that are practicable and can map the course of viral disease and recovery for detailed control diagnostics. According to Friman and Wesslén (2000), fever (>38°C or 0.5–1° higher than usual) and an increased resting heart rate (>10 bpm higher than normal) are contraindications for physical training. Therefore, deciding on the right time to restart training is essential (Dores and Cardim, 2020). Long breaks in physical training are critical for performance development in elite athletes; too short breaks in the case of viral infection can cause a relapse and incomplete curing of an infection can lead to severe health risks (Verwoert et al., 2020).

The scientific community is working on the topic of how to optimize the time to return to sports after a viral infection and which diagnostic indicators could support decision making (Dores and Cardim, 2020; Nieß et al., 2020; Schnellhorn et al., 2020; Verwoert et al., 2020). Viral infections affect the cardiovascular system and lead to an increased resting heart rate (HR) in the presence of fever (Karjalainen and Viitasalo, 1986). Acute upper respiratory tract infections (URTIs), including influenza, respiratory syncytial virus, and bacterial pneumonias, are well-recognized triggers for cardiovascular diseases (Cowan et al., 2018). The emergence of SARS-CoV-2 has rapidly grown into a pandemic and practical parameters for daily health monitoring are necessary. A large proportion of affected patients have been reported to have underlying cardiovascular diseases, and myocardial infarctions were noted to occur after an infection (Madjid et al., 2020). A recent case study on cardiovascular changes occurring during an infection with COVID-19 shows that heart rate variability (HRV) decreases but HR does not increase at rest (Buchhorn et al., 2020). Thus, it is not sufficient to only examine resting HR, but further parameters are required for a differential analysis.

For athletes and coaches, it is essential to perform detailed health monitoring in conjunction with performance monitoring to ensure a very early detection of a viral infection and to guarantee a safe return to training (Hagen et al., 2020). For the control of physical load, athletes often use HR and HRV measurements to individualize the training load and to detect symptoms of overtraining at an early stage (Uusitalo et al., 2000; Buchheit, 2014; Plews et al., 2014; Hottenrott and Hoos, 2017). To the best of our knowledge, no study has examined HR and HRV measurements in elite athletes to monitor health status during and after a viral infection and to individualize return to training.

Cardiac vagal control reflects the activity of the vagus nerve regulating cardiac functioning and can be inferred via HRV measurements (Laborde et al., 2018; Schneider et al., 2018). Most often, HRV analysis is derived from resting data in a single supine or sitting position. However, some studies show that RR measurements in a single resting position are not sufficient to detect training-induced fatigue in athletes (Buchheit, 2014; Plews et al., 2014; Bellenger et al., 2016a,b). A passive head-up tilt test in supine and upright positions results in specific changes in the spectral characteristics of HRV as a result of reduced vagal and enhanced sympathetic outflow and gives valuable indications that a change of body position can lead to additional information about regulation patterns of the cardiac autonomic system (Tulppo et al., 2001). An active switching from the supine to the upright position imposes stress by gravitational pooling of the blood in the splanchnic venous reservoir and leg veins (Stewart et al., 2006). Consequently, the autonomic nervous system is required to maintain the hemodynamic to avoid cerebral hypoperfusion. From supine to standing, HR increases (RR intervals become shorter) and high frequency power (parasympathetic) is depressed compared to supine, whereas low frequency power (partially sympathetic) increases (Aubert et al., 2003).

An orthostatic test (OT) provides a practical method for the detection of overload and overtraining in athletes and can be used to assess the autonomic nervous system's response to physical exercise and training (Le Meur et al., 2013; Buchheit, 2014; Plews et al., 2014; Schneider et al., 2019; Barrero et al., 2020). In addition, changes in body position can provoke specific responses in HR dynamics and could therefore provide more specific information about autonomic nervous system regulation patterns (Tulppo et al., 2001). During orthostatic tolerance assessment, HRV patterns in both supine and standing positions are affected by the different involvement of cardiopulmonary receptors, i.e., cardiac preload, and hence, tuned changes in plasma volume and/or peripheral vasomotor tone. Among other factors, these parameters likely support changes in autonomic patterns and HRV also during different training loads and phases (Schmitt et al., 2015). For precise analysis, monitoring in athletes should not be limited to the measurement of cardiac autonomic function in just one body position but should consider assessing the response in two different body positions (e.g., supine and standing) (Buchheit, 2014; Schmitt et al., 2015; Ravé and Fortrat, 2016; Hottenrott and Hoos, 2017; Hottenrott et al., 2019).

**Figure 1:**
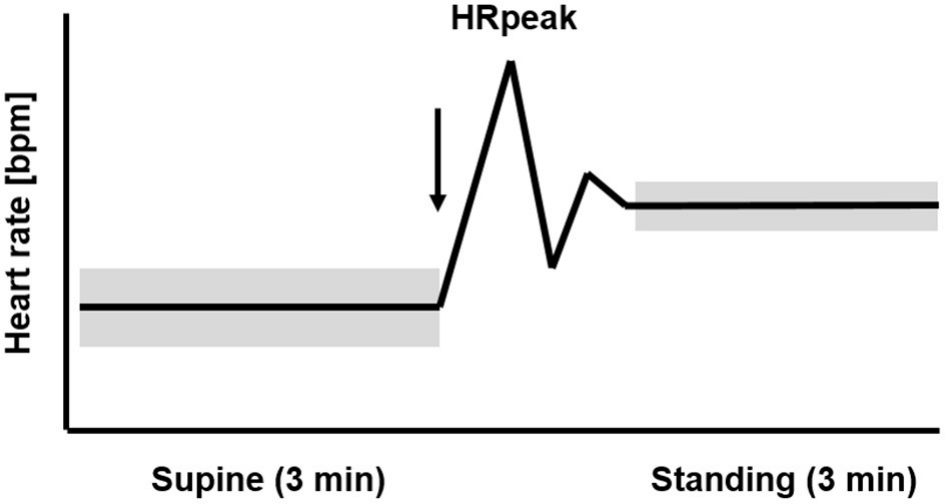
Temporal course of the heart rate (HR) and heart rate variability (HRV) during 3 min supine position followed by 3 min standing position (orthostatic test, including HRpeak) in healthy athletes with uncompromised performance. The arrow marks the change in body position from supine to standing up. The widths of the bars represent the higher HRV in the supine than the standing position (mod. Hottenrott and Hoos, 2017).

Figure 1 displays the temporal course of the heart rate in healthy athletes with uncompromised performance during the OT. The HR is low in the supine position and rises rapidly during active standing-up. In the supine position, the efferent vagal activity often calculated with the parameter root mean square of successive differences (RMSDD) is much higher than in the standing position (Bellenger et al., 2016a). After active standing-up, the HR will reach the first peak (HRpeak) after 15–20 s (Flachenecker, 2000; Rooke and Sparks, 2003). The rate of heart rate increase from rest to exercise or supine to standing is another parameter providing information about the cardiac autonomic system to detect training-induced fatigue (Nelson et al., 2014; Bellenger et al., 2016a,c). Thereafter, a counter-regulation with a rapid decrease of the HR occurs, before the HR rises again and stabilizes in the standing position. When there is high circulation stability, HR in the standing position remains higher than the HR in the supine position. The average HR in the standing position of healthy and non-fatigued athletes is on average 10–20 bpm higher than the HR at rest in the supine position (Aubert et al., 2003; Hottenrott and Hoos, 2017). Standing also induces a three- to four-fold decrease of vagal HRV indices compared to the supine position (Aubert et al., 2003; Hottenrott and Hoos, 2017).

Viral diseases (e.g., influenza, Epstein-Barr virus, coronavirus) have different individual progressions (Polak et al., 2020) and can lead to considerable risks/long-term consequences. Therefore, it is not suitable to give general recommendations on time off from training for athletes. The severity of the disease and the course of the recovery process can be very individual. In athletes, viral diseases are usually mild and are often not taken seriously, although considerable risks/long-term consequences can nevertheless occur if training is continued during the infection (Roberts, 1986). Regarding daily monitoring with the OT, it is expected that a viral infection with the presence of fever will lead to an increase in supine resting heart rate and affect the overall cardiac autonomic system (Karjalainen and Viitasalo, 1986; Polak et al., 2020).

It is the aim of the present case report to display how the OT, which is based on the detection of RR intervals in heart rate time series in supine and standing positions, can monitor the progression and recovery process before, during, and after a viral disease in a high-performance endurance athlete providing immediate day-to-day feedback about autonomic nervous system recovery status.

## Methods

### Participant

The participant is a 30-year-old male elite marathon runner with a personal best time in the marathon of 2:18 h. He has competed in the sport of running since he was 13 years. He has a resting HR of 47 bpm, a maximal HR of 188 bpm, and a VO_2_max of 71 ml/kg/min and receives medical clearance in a sports medical check-up annually. The athlete's training is monitored routinely throughout the entire year and he performs an OT daily (Figure 1). The participant contracted an upper respiratory tract infection (URTI, diagnosed by a physician) 4 weeks after running a marathon race in 2:21 h. He had intense flu symptoms for 6 days and fever between 38.5° and 39.3°C for 3 days on days 13, 14, and 15; he did not take any fever-lowering medication. Clinical diagnostics were not accompanied by laboratory diagnostics.

The participant provided informed consent in accordance with the institutional review board and the guidelines of the Helsinki World Medical Association Declaration.

### Measurements

Ten days before (pre), during, and 10 days after (post) the viral infection, a daily (after a night's sleep) orthostatic test (OT) and a position change test with 3 min in the supine and 3 min in the standing position were performed (Bourdillon et al., 2017; Hottenrott and Hoos, 2017). The recordings were taken in the supine position without a given breathing rhythm after the morning visit to the restroom. The OT was performed with continuous beat-to-beat recording of the heart rate (RR measurement with V800 and H10 sensor, Polar Electro GmbH, Finland, Gilgen-Ammann et al., 2019). The calculated parameters of the OT included HR and the vagal time-domain HRV parameter RMSSD in supine and standing positions as well as the peak HR (HRpeak) and time to HR peak (tHRpeak) in the standing position. Daily the athlete recorded his training and recorded information about his state of health, especially about symptoms of illness.

### Data Analysis and Statistics

The calculation of the RR data from the OT was performed with the Kubios HRV Premium Software (Version 3.4.1, Tarvainen et al., 2014). The RMSSD was calculated for 2 min in the supine and 2 min in standing position. The first of the 3 min in the supine position was not used for data analysis but served for physiological stabilization (Bourdillon et al., 2017; Hottenrott and Hoos, 2017). The smallest worthwhile change (SWC) in standing RMSSD from baseline was deemed as 0.5 of the individual baseline coefficient variation (CV) (Plews et al., 2013; Buchheit, 2014) from the baseline during average training load at sea level in a healthy state over 3 month prior to the viral infection (as a fixed reference point). Differences in HR, RMSSD, HRpeak, and tHRpeak between the measure points (days) were evaluated. Statistical analysis was performed with the software SPSS 25.0 (IBM Statistics, USA) for Windows. Prior to the analysis of the differences between 10 days before, during, and 10 days after the upper respiratory tract infection (URTI), Gaussian distribution of the data was verified by the Shapiro–Wilk test. A single factor repeated measures ANOVA was used to test whether there were statistical differences between the mean values of the respective parameters over the three measurement points (pre, URTI, post). For statistical analysis, ANOVA with *post-hoc* multiple comparisons and Bonferroni correction was applied. The sphericity was determined in advance using the Mauchly test. The data are presented as mean ± standard deviation. The level of significance was set at 0.05 (*p* < 0.05).

## Results

**Figure 2:**
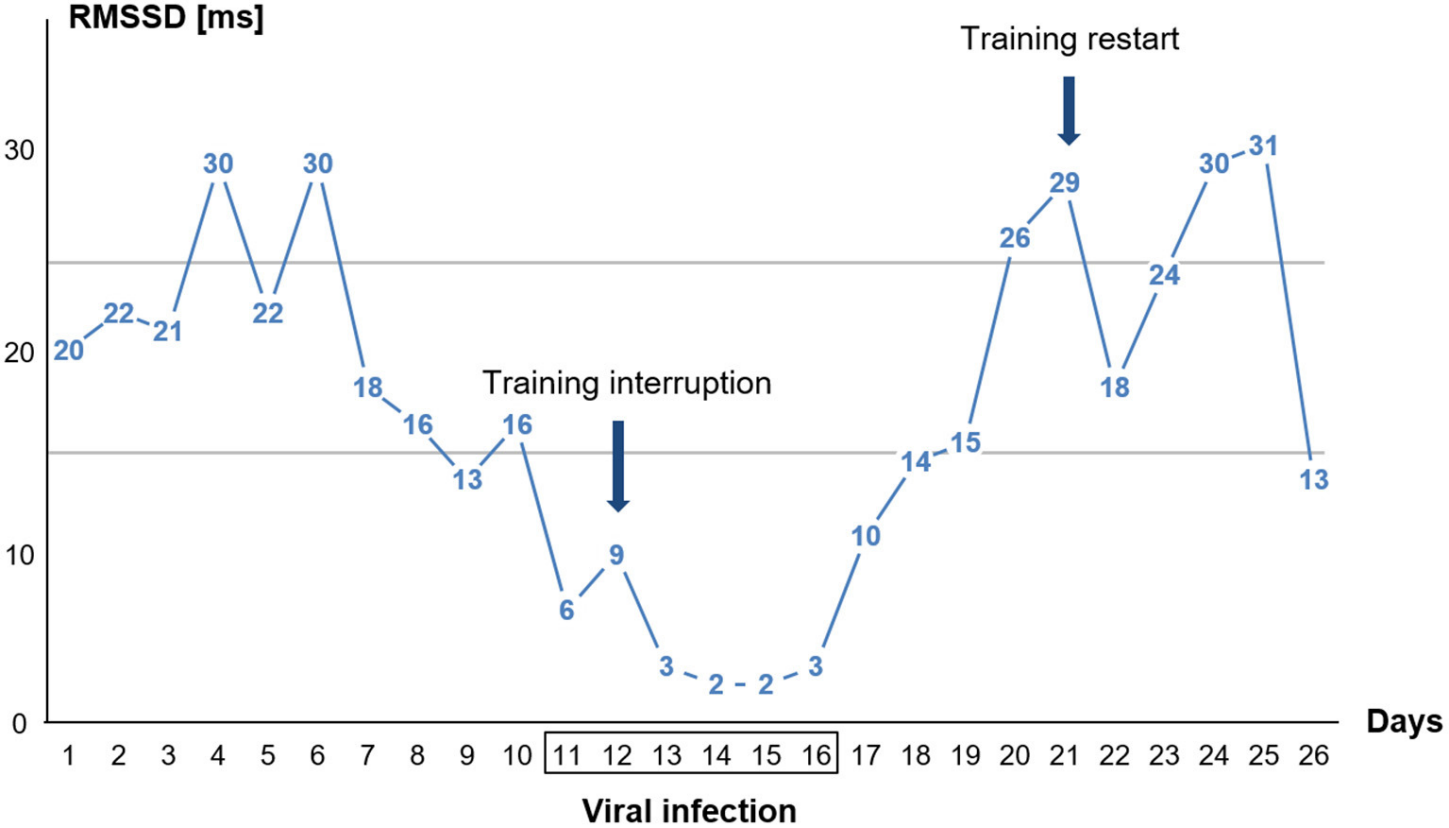
Single day values for time-domain heart rate variability (HRV) parameter RMSSD in the standing position of the orthostatic test (OT) before, during, and after the viral infection. The athlete had fever on days 13, 14, and 15. The two horizontal lines indicate the area of the individual smallest worthwhile change (SWC) in standing RMSSD during baseline (see section Methods).

The athlete maintained his average training load of 14–21 km of easy to moderate running per day on days 1–11. He had 1 day of rest on day 9. The athlete was absent from training on day 12–20 and restarted training on day 21 with an easy 10 km run (aerobic) followed by 2 days of easy running training of 8–12 km, on day 24, he completed 20 km of easy running, and on day 25, he did no running but did 3 h of biking. The ANOVA analysis shows significant main time effects for HR supine [*F*_(2.10)_ = 21.913, *p* < 0.001, partial η^2^ = 0.814], HR standing [*F*_(2.10)_ = 47.253, *p* < 0.001, partial η^2^ = 0.904], HRpeak [*F*_(2.10)_ = 22.586, *p* < 0.001, partial η^2^ = 0.819], tHRpeak [*F*_(2.10)_ = 38.261, *p* < 0.001, partial η^2^ = 0.819], RMSSD supine [*F*_(2.10)_ = 5.625, *p* = 0.023, partial η^2^ = 0.529], and RMSSD standing [*F*_(2.10)_ = 21.761, *p* < 0.001, partial η^2^ = 0.813]. *Post-hoc* comparisons showed that during the 6-day viral infection period, all values, except the RMSSD value in the supine position, changed significantly from the previous 10-day pre-measurement (Table 1). The HR increased by an average of 11 bpm in the supine position and by 22 bpm in the standing position, the HRpeak by 13 bpm, and the tHRpeak increased by 18 s. The RMSSD in the standing position decreased from 20.8 ms to 4.2 ms and showed the largest changes over the course of the viral infection days (Table 1; Figure 2).

**Table 1:** Values (mean ± SD) of HR, RMSSD, HRpeak, and tHRpeak (time from HR in the supine position until HRpeak in the standing position) in the orthostatic test (OT) 10 days before, during, and 10 days after the upper respiratory tract infection (URTI) from a 30-year-old elite marathon runner.

**Position**	**Parameter**	**Pre**	**URTI**	**Post**	**Pre-URTI**	**URTI-post**	**Pre-post**
		**days 1–10**	**days 11–16**	**days 17–26**	***p*-value**	***p*-value**	***p*-value**
2 min supine	HR [bpm]	55.5 ± 2.8	66.0 ± 7.2	52.1 ± 3.2	0.013	0.013	0.070
	RMSSD [ms]	18.0 ± 9.2	11.5 ± 7.6	31.4 ± 20.3	0.595	0.005	0.65
2 min standing	HR [bpm]	86.0 ± 4.6	107.7 ± 9.2	79.0 ± 6.0	0.001	0.002	0.178
	RMSSD [ms]	20.8 ± 5.7	4.2 ± 2.8	21.0 ± 7.9	0.002	0.041	0.307
Supine-standing	HRpeak [bpm]	93.4 ± 4.5	106.3 ± 9.4	88.4 ± 4.7	0.012	0.010	0.184
	tHRpeak [s]	16.8 ± 2.2	35.0 ± 16.2	18.9 ± 3.5	0.003	0.008	0.124

The 10-day post-measurements differed significantly in all parameters on average from the 6-day viral infection period. The HR in the supine position decreased by 14 bpm, the HR in the standing position by 19 bpm, and the HRpeak by 18 bpm. The RMSSD values increased by 20 ms (supine) and 17 ms (standing), and the HR ascended faster again. The 10-day post RMSSD values in the supine and standing positions and the tHRpeak did not differ from the 10-day pre-measurements (Figure 2).

The graph in Figure 2 displays that the RMSSD values were already decreasing days before the viral infection symptoms were felt. Training was then stopped when the viral infection symptoms (sore throat) appeared (day 12). Upon relief of the viral infection symptoms, RMSSD levels immediately increased. The athlete restarted his training (day 21) after the RMSSD values had approximately reached the initial values before the illness.

**Figure 3:**
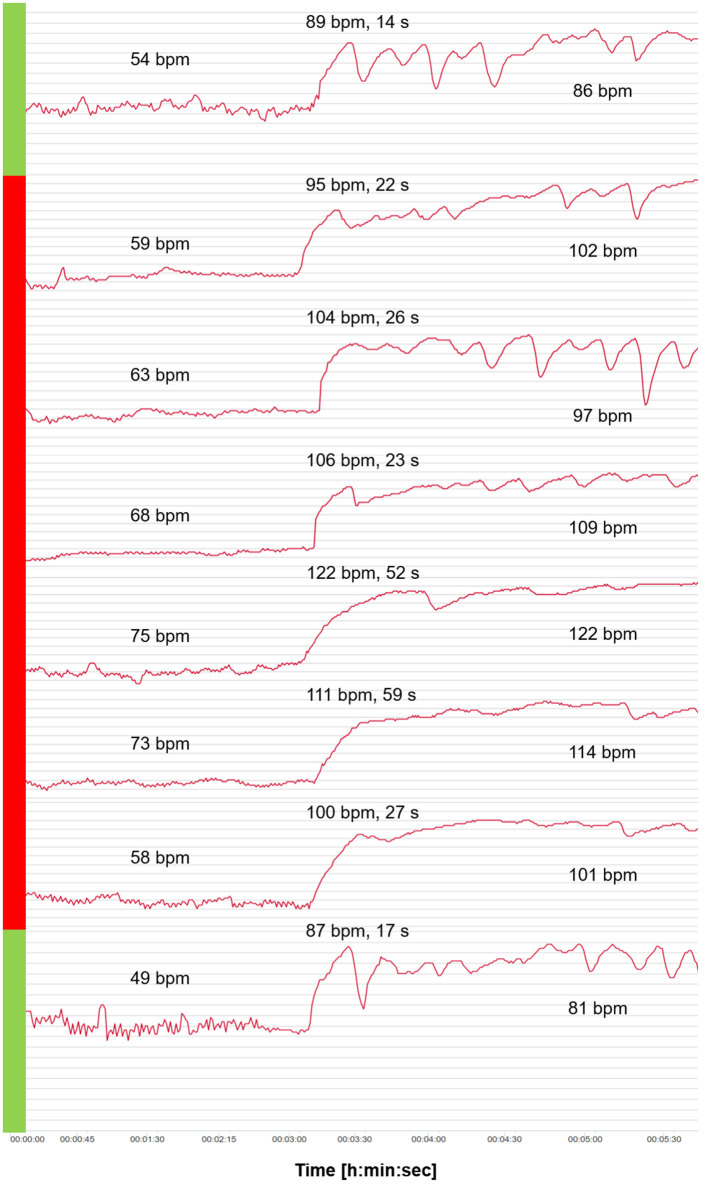
Heart rate (HR) curves from the orthostatic test (OT) for the 6-day viral infection period (red) and a curve before and after the viral infection period (green). Mean values of HR analyzed after 2 min in the supine position, and HRpeak, tHRpeak, and mean values of HR analyzed after 2 min in the standing position.

Figure 3 shows the HR curves from OT for the 6-day viral infection period and a typical curve before and after the viral infection period. An increase in HR values in supine and standing positions as well as 2 days with extremely slow tHRpeaks (52 s and 59 s) were apparent, and counter-regulation was missing. On the days of the slow tHRpeak (days 4 and 5 of vthe iral infection), the HRpeak reached the highest values (122 bpm and 111 bpm).

## Discussion

The present case study is the first to show the interaction between a viral infection and the daily monitoring of the cardiac autonomic control in an elite athlete. The case study is particularly unique due to the fact that the data are ecologically valid (not lab-based), and obtained from an elite endurance athlete.

The main finding is that a viral infection had a direct influence on HR and HRV. An increase in HR was accompanied by a decrease in RMSSD in the OT in the standing position. It seems that cardiac vagal activity is suppressed in the presence of a viral infection, which is also the case in patients with fever (Lin et al., 2006; Carter et al., 2014). An increase in resting HR and a decrease in RMSSD suggests a suppression of parasympathetic activity (Buchheit, 2014; Laborde et al., 2018). The kinetics of HR from supine to standing was significantly different in the HR increase (tHRpeak) from a typical course in a healthy condition. With the onset of the viral infection symptoms, there was a slower increase, which was particularly pronounced after 3 days of the 6-day period. The state of research according to changes in cardiac autonomic regulation during viral infection strengthen the evidence that specific measures of HRV are valid indicators of cardiac autonomic responsiveness (Malik et al., 2019). Bellenger et al. (2016c) found a slower HR acceleration at the onset of exercise in athletes suffering from exercise-induced fatigue (overload training). A reduced performance capacity of the athletes and altered cardiac autonomic control due to fatigue may also apply to a viral infection. In the context of viral diseases, HR increase in regard to a change in body position has not been investigated so far. A small number of studies conducted with endurance athletes demonstrated a decrease in HR acceleration following overload training, indicating it may be a potential indicator of training-induced fatigue (Nelson et al., 2014; Bellenger et al., 2016a).

The second main result is that HR and HRV values changed more substantially in the standing position than in the supine position during viral infection. This was particularly pronounced in the RMSSD values, which decreased from 20 ms before to 4 ms during viral infection. The cardiac autonomic system reacted more sensitively in the standing position compared to the supine position in the present data. The RMSSD changed significantly from pre to viral infection values in the standing position only (Table 1). The analysis of the single day values for the RMSSD in the standing position shows that the athlete stopped training when the flu symptoms appeared. However, according to the HRV analysis, a training suspension 2–3 days earlier might have been preferable to avoid weakening the immune system further (Figure 2). The return to training was reasonably chosen after the HRV values had returned to the initial level and were elevated for several days. The further fluctuations of the post-values of the 6-day viral infection period are related to the training process. Moderate aerobic training (low-intensity training) leads to better values in RMSSD compared to pre-values due to positive effects on vagal activity (increases in parasympathetic activity) (Stanley et al., 2013). The reason for the strong decrease in RMSSD values on day 26 could be the more intensive training the day before (training phases with increased time spent at high intensity suppress parasympathetic activity) (Buchheit and Gindre, 2006; Plews et al., 2014; Schneider et al., 2019). This might indicate that the performance capacity and health status level for high training stimuli was not yet given.

## Limitations

We chose morning resting HRV recordings due to the practicality of the measurement and with a focus on the vagal HRV parameter RMSSD because this parameter provides valid results in longitudinal analysis without a given breathing rate in the context of training and health status, which cannot be guaranteed using frequency-domain analysis of HRV (e.g., low frequency and high frequency power) without a given breathing control (Nakamura et al., 2015).

Furthermore, it could be shown that RMSSD determined in a short period of already 1 min (calculation of 1 min after 1 min stabilization period) is sensitive to training-induced changes in athletes, and can be used to track cardiac autonomic adaptations (Nakamura et al., 2015). In regard to OT, Schäfer et al. (2015) found no differences using 2 min intervals compared to 4 min intervals in both supine and standing positions for monitoring training and recovery processes. Therefore, the chosen procedure of a 3-min recording time in supine and standing positions with analyzed intervals of 2 min respectively should provide representative values of HR and RMSSD.

## Conclusion

The findings of this case report have some implications for sports practitioners and coaches looking to both ensure the health of their athletes, and for using HRV as a tool to monitor training process and the return to sport after a viral infection. For endurance athletes, a control by means of resting HR in one body position does not seem to be sufficient. Accordingly, the data have provided supportive rationale as to why the OT with a change from supine to standing body position and the detection of different indicators based on HR and a vagal driven time-domain HRV parameter (RMSSD) is likely to be useful to detect viral diseases early on when implemented in a daily routine. Given the case study nature of the findings, future research has to be conducted to investigate whether the use of the OT might be able to offer an innovative, non-invasive, and time-efficient possibility to detect and evaluate the health status of (elite endurance) athletes.

## Data Availability Statement

The original contributions presented in the study are included in the article/supplementary material, further inquiries can be directed to the corresponding author/s.

## Ethics Statement

Ethical review and approval was not required for the study on human participants in accordance with the local legislation and institutional requirements. The patients/participants provided their written informed consent to participate in this study. Written informed consent was obtained from the individual(s) for the publication of any potentially identifiable images or data included in this article.

## Author Contributions

LH: conceptualization, methodology, investigation, writing—original draft preparation, and project administration. LH, KH, and TG: validation. LH and KH: data curation and visualization. LH, KH, TG, TW, and AF: writing—review and editing. AF: supervision. All authors have read and agreed to the published version of the manuscript.

## Conflict of Interest

The authors declare that the research was conducted in the absence of any commercial or financial relationships that could be construed as a potential conflict of interest.

## Publisher's Note

All claims expressed in this article are solely those of the authors and do not necessarily represent those of their affiliated organizations, or those of the publisher, the editors and the reviewers. Any product that may be evaluated in this article, or claim that may be made by its manufacturer, is not guaranteed or endorsed by the publisher.
